# Comparative cytocompatibility of the new calcium silicate-based cement NeoPutty versus NeoMTA Plus and MTA on human dental pulp cells: an in vitro study

**DOI:** 10.1007/s00784-022-04682-9

**Published:** 2022-09-02

**Authors:** Ana Lozano-Guillén, Sergio López-García, Francisco Javier Rodríguez-Lozano, José Luis Sanz, Adrián Lozano, Carmen Llena, Leopoldo Forner

**Affiliations:** 1 Department of Stomatology, Faculty of Medicine and Dentistry, Universitat de València, 46010 Valencia, Spain; 2 Hematopoietic Transplant and Cellular Therapy Unit, Instituto Murciano de Investigación Biosanitaria Virgen de La Arrixaca, IMIB-Arrixaca, University of Murcia, 30120 Murcia, Spain; 3 Department of Dermatology, Stomatology, Radiology and Physical Medicine, Morales Meseguer Hospital, Faculty of Medicine, University of Murcia, 30008 Murcia, Spain

**Keywords:** Cytocompatibility, Biocompatibility, Vital pulp therapy, Calcium silicate cements, In vitro

## Abstract

**Objectives:**

The aim of the present in vitro study is to determine the cytocompatibility of the recently introduced NeoPutty in contact with human dental pulp cells compared with its precursor NeoMTA Plus and the classic gold standard MTA Angelus.

**Materials and methods:**

Sample disks were obtained for each of the tested materials (5 mm diameter; 2 mm thickness; *n* = 30), along with 1:1, 1:2, and 1:4 material eluents. HDPCs were extracted and cultured with the tested materials (test groups) or in unconditioned medium (control group), and the following biocompatibility assays were performed: MTT assay, scratch wound assay, cell cytoskeleton staining assays, and cell attachment assessment via SEM. Additionally, material ion release and surface element composition were evaluated via ICP-MS and SEM–EDX, respectively. Each experimental condition was carried out three times and assessed in three independent experiments. Statistical significance was established at *p* < 0.05.

**Results:**

1:2 dilutions of all the tested materials exhibited a comparable cell viability to that of the control group at 48 and 72 h of culture (*p* < 0.05). The same was observed for 1:4 dilutions of the tested materials at 24, 48, and 72 h of culture (*p* > 0.05). All the tested materials exhibited adequate cytocompatibility in the remaining biocompatibility assays. MTA exhibited a significantly higher calcium ion release compared to NeoPutty and NeoMTA Plus (*p* < 0.05).

**Conclusion:**

The results from the present work elucidate the adequate cytocompatibility of NeoPutty, NeoMTA Plus, and MTA Angelus towards human dental pulp cells.

**Clinical relevance:**

Within the limitations of the present in vitro study, our results may act as preliminary evidence for its use in vital pulp therapy as a pulp capper. However, results need to be interpreted with caution until further clinical supporting evidence is reported.

## Introduction

Vital pulp therapy (VPT) embraces a series of conservative procedures [1] that aim to treat the dentin-pulp complex that has been injured due to dental trauma, carious lesions, iatrogenic events, or during restorative treatment [2]. VPT is indicated in those cases where there are signs and/or symptoms of reversible or even irreversible pulpitis, and no periapical lesions of endodontic origin are present [3]. It includes, from lower to higher degree of invasiveness, the following: indirect pulp capping, direct pulp capping, and pulpotomy [3, 4]; root canal treatment (RCT) should be avoided whenever a VPT approach is possible, since the latter depends on a physiological response and a biologically based outcome instead of the extirpation of potentially viable pulp tissue and the subsequent local response surrounding tissues towards root canal filling materials [5].

In VPT procedures, pulp capping agents or pulp cappers are used. Pulp cappers are a subgroup of dental materials which are capable of inducing the formation of a mineralized layer that protects the dentin-pulp complex and establish a biocompatible medium for it to repair, to maintain the tooth’s vitality [6]. For decades, the gold standard material used for such purpose in VPT has been mineral trioxide aggregate (MTA) due to its desirable biological properties like biocompatibility, bioactivity/biomineralization, low solubility, and hydrophilicity [7]. However, it is not exempt from disadvantages, such as its handling difficulty and long setting time [8]. Nevertheless, MTA, as a Portland cement-based material, served as the precursor of novel bioceramic or calcium silicate-based material compositions with enhanced biological properties and antibacterial activity [9]. Ideally, such properties need to be tested in vitro before their clinical application, using cellular populations as a means of anticipating their behavior when placed in contact with biological tissues.

Among Portland cement-based materials, the most widely used is MTA Angelus (Angelus, Londrina, Brazil), which is a “white” MTA composition presented in a powder-liquid format. Water is used as a vehicle, and the powder formed by tricalcium silicate, dicalcium silicate, tricalcium aluminate, silicon oxide, potassium oxide, aluminum oxide, sodium oxide, iron oxide, calcium oxide, bismuth oxide, magnesium oxide, and insoluble residues of crystalline silica [10].

Another tricalcium silicate-based material is NeoMTA Plus (Avalon Biomed Inc., Bradenton, USA) which is also presented in a powder-liquid format: the powder contains tricalcium silicate, dicalcium silicate, and tantalum oxide, and the liquid contains water and polymers [11]. Interestingly, NeoMTA Plus incorporates tantalum oxide as a radiopacifier, instead of bismuth oxide, which has been described to be responsible for the discoloration caused by the classic MTA [12].

Most recently, a new version of NeoMTA Plus has been introduced into the market as NeoPutty (Avalon Biomed Inc., Bradenton, USA). As opposed to its precursor, this new material comes in a premixed format and is composed of the following: tantalum oxide, tricalcium silicate, calcium aluminate, dicalcium silicate, tricalcium aluminate, and calcium sulfate. Due to its recent commercialization, there is not much evidence about the cytotoxicity of this material [13]. However, in vitro studies that assess the biocompatibility of materials, especially those which will be placed in direct contact with vital pulp tissue, are necessary to evaluate their potential risks and adequacy for treatment [14].

Human dental pulp cells (hDPCs) are involved in the process of reparative dentinogenesis. For this reason, one of their experimental applications is dentin-pulp complex repair/regeneration via cell-based and tissue engineering approaches [15, 16]. Furthermore, it has been described that both calcium silicate-based materials and Portland cement-based materials are able to interact with these cells and aid with their osteo/odontogenic differentiation, proliferation, and attachment [17].

Accordingly, the present in vitro study aimed to determine the cytocompatibility of the recently introduced NeoPutty in contact with human dental pulp cells compared with its precursor NeoMTA Plus and MTA Angelus.

## Materials and methods

### Tested materials and extract preparation

MTA (Angelus, Londrina, PR, Brazil), NeoMTA Plus (NuSmile Avalon Biomed, Bradenton, FL, USA), and NeoPutty (NuSmile Avalon Biomed) were tested in this study. The material compositions, manufacturers, and lot numbers are presented in Table 1. Material specimens (diameter = 5 mm, thickness = 2 mm) were prepared using custom-made polyoxymethylene molds, and left undisturbed to set at 37 °C in 5% CO_2_ environment and 95% relative humidity for 48 h. Once set, the surfaces of the specimens were sterilized for 20 min to ultraviolet light. Accordingly, ISO 10993–12 was used to evaluate the cytotoxicity effects of each group. The final concentrations were 1:1, 1:2, and 1:4.

**Table 1:** The material compositions, manufacturers, and lot numbers

Materials	Manufacturer	Composition	Lot number
NeoMTA Plus	NuSmile Ltd (Avalon Biomed). 3315 West 12th Street Houston, TX 77008 USA	**Powder:** tricalcium silicate, dicalcium silicate, tantalum oxide, and minor amounts of calcium sulfate and tricalcium aluminate **Liquid:** water and proprietary polymers	2019091001
NeoPutty	NuSmile Ltd (Avalon Biomed). 3315 West 12th Street Houston, TX 77008 USA	Tantalite, tricalcium silicate, calcium aluminate, dicalcium silicate, tricalcium aluminate, calcium sulfate, proprietary organic liquid and stabilizers	2020071501
MTA Angelus	Angelus. Rua Waldir Landgraf, 101 Bairro Lindóia CEP 86031–218, Londrina, PR Brasil	Tricalcium silicate, dicalcium silicate, tricalcium aluminate, calcium oxide, calcium tungstate	101752

### Ion release of tested materials

Each of the tested materials was placed in deionized water (Milli-Q; Merck KGaA, Darmstadt, Germany) and their ion release was evaluated using an inductively coupled plasma-optical emission spectrometry (ICP-MS). Three specimens for each material were prepared for this purpose. The proportion of aluminum (Al), silicon (Si), sulfur (S), calcium (Ca), strontium (Sr), zirconium (Zr), barium (Ba), and tungsten (W) released from each material was analyzed at day 1 in triplicate, and the elements were calibrated with pure deionized water. Analyses were performed independently in triplicate (*n* = 3).

### Extraction of third molars and isolation of hDPCs

HDPCs were isolated from impacted third molars (age 15–25; *n* = 10), extracted for orthodontic reasons. A previous written informed consent was obtained from every patient through the University of Murcia/School of Dentistry, with the approval of its ethics committee (ID: 2543/2019). Human dental pulps were obtained from the pulp chamber and root canals of the extracted third molars by means of a barbed broach. Then, the pulp was rinsed with Hank’s Balanced Salt Solution (Gibco BRL, Burlingame, CA) and digested using 3 mg/mL collagenase A (Sigma-Aldrich, St Louis, MO). The resultant cells were subsequently cultured in basal medium containing alpha modified minimum essential medium (α-MEM, Lonza, UK) supplemented with 10% fetal calf serum (FCS, Lonza, UK), 2 mM L-glutamine, and a mix of 100 units/mL penicillin with 100 μg/ml streptomycin at 37 °C and 5% CO_2_. Cells were passaged when approaching 80% confluency, and cells from passages 2–4 were used for this study.

### MTT assay

Assessment of the metabolic activity of hDPCs treated with material eluates was performed using a colorimetric 3-(4,5-dimethylthiazol-2-yl)-2,5-diphenyltetrazolium bromide (MTT) assay as previously described [18]. Cell metabolic activity was examined at 24, 48, and 72 h of culture. Material eluates (1:1; 1:2; 1:4) were placed in direct contact with the hDPC culture, and an MTT reagent (Sigma-Aldrich) was added for 4 h as specified by the manufacturer’s instructions. One hundred milliliters/well dimethylsulfoxide (Sigma-Aldrich) was then added to dissolve the formazan crystals. Covered plates were kept in the dark for 2–4 h. Afterwards, the formazan production was transferred to the spectrophotometer (ELx800; Bio-Tek Instruments, Winooski, VT), and the metabolic activity was analyzed at a wavelength of 570 nm.

### Cell migration evaluation (scratch wound assay)

HDPCs from passages 2–4 were seeded at a concentration of 2 × 105 cells in a 12-well plate. After 48 h, a scratch wound was performed in the cell monolayer with a sterile 100-μl pipette tip and exposed to the material extracts or control group (medium without material extracts). Cell migration distances were assessed at three time intervals: first 24-h period (0–24 h), second 24-h period (24–48 h), and third 24-h period (48–72 h). To account for width variations among the scratch wounds, migration rates were presented as percentage areas of relative wound closure or RWC and calculated as follows: RWC (%) = (wound closure area (pixels)/total number of pixels) × 100. Results were measured as the percentage of the total wound area at the different time points relative to the total wound area at 0 h for each respective well and ImageJ software (National Institutes of Health, Bethesda, MD, USA) was used to measure the percentage of open wound area at each time point, relative to the same wound area at 0 h in the same well. Four standardized points were evaluated.

### Cell cytoskeleton staining assays

Fluorescent-phalloidin labeling was used to evaluate the organization of the F-actin and possible changes in cell morphology. Briefly, 3 × 104 cells were added on glass coverslips, allowed to adhere and spread, and cultured in complete growth medium alone (control) or material extracts for 72 h at 37 °C. Then, HDPCs were fixed with 4% paraformaldehyde for 15 min and permeabilized with 0.05% Triton X-100 for 10 min at room temperature. Then, cells were washed twice with PBS, and cell F-actin cytoskeleton and nuclei were then stained with Invitrogen™ AlexaFluor™594-labeled phalloidin (Thermo Fisher Scientific) and 4,6-diamidino-2-phenylindole dihydrochloride (DAPI) (Thermo Fisher Scientific), respectively, at r/t in the dark for 30 min. Finally, the representative images were captured using the Leica TCS SP2 confocal microscope (Leica, Wetzlar, Germany). Three different pictures were captured in random fields.

### Scanning electron microscopy (SEM) analyses

Eighteen 2 mm-high and 5 mm diameter disks of the tested materials were randomly divided into three groups (*n* = 6 samples/group) and used to evaluate the hDPC attachment to the surface of the materials. Briefly, a total of 5 × 10^4^ hDPCs were seeded onto each disk and cultured for 3 days. Then, the specimens were rinsed with PBS, fixed for 4 h in the refrigerator, and treated with a series of solutions with an ascending proportion of alcohol, up to 100% to dehydrate samples. Specimens were mounted on brass stubs and sputter-coated with 5 nm of gold. Finally, images were randomly taken at different areas of each specimen at 100 × , 300 × , and 1500 × magnification. Three specimens were sputter-coated with carbon, and the surfaces were examined by a SEM microscope (SEM Jeol 6100 EDAX, Tokyo, Japan) coupled with an energy-dispersive X-ray spectroscopy system (EDX; Oxford INCA 350 EDX, Abingdon, UK) with operating conditions of 20 kV. The full scale for quantification was 8677 cts.

### Statistical analysis

Each experimental condition was carried out three times and assessed in three independent experiments. Data was expressed as mean ± standard deviation (SD). The homogeneity of variance and normal distribution of the data were confirmed. Consequently, a parametric analysis was performed by ANOVA multiple comparisons test with Tukey modification using SPSS software (IBM Analytics, version 21). Non-significant (NS): *p* > 0.05, *p < 0.05, **p < 0.01, and ***p < 0.001.

## Results

### Ion release

Ion release from each of the tested materials, measured with ICP-MS, is shown in Table 2. Zirconium (Zr) was detected in all groups, whereas sulfur (S) was not found in MTA Angelus. MTA Angelus showed a higher release of Ca^2+^ and Strontium (Sr) (*p* < 0.05), while aluminum (Al) and barium (Ba) ion release were significantly increased in NeoMTA Plus (*p* < 0.05). In contrast, NeoPutty had the lowest release of Ba and Sr ions (*p* < 0.05).

**Table 2:** ICP-MS analysis of tested materials

Sample name	27 Al [He]	29 Si [He]	34 S [He]	42 Ca [He]	88 Sr [He]	91 Zr [He]	137 Ba [He]
	Conc. [ppb]	Conc. [ppm]	Conc. [ppm]	Conc. [ppm]	Conc. [ppb]	Conc. [ppm]	Conc. [ppb]
NeoPutty	47.58 ± 0.04^AB^	4.17 ± 0.00	0.93 ± 0.02^AB^	10.55 ± 0.00^B^	28.40 ± 0.01^AB^	0.27 ± 0.04^AB^	0.72 ± 0.00^AB^
NeoMTA Plus	115.72 ± 0.02^AC^	6.75 ± 0.02	7.80 ± 0.02^AB^	9.04 ± 0.02^C^	425.40 ± 0.02^AC^	0.15 ± 0.03^A^	5.23 ± 0.00^AC^
MTA Angelus	< 0.000 ± 0.00^AC^	5.84 ± 0.00	< 0.000 ± 0.00^AC^	79.10 ± 0.03^BC^	1815.25 ± 0.02^BC^	0.14 ± 0.00^B^	2.39 ± 0.00^BC^

Uppercase A (^A^) indicates significant difference (*p* < 0.05) between NeoPutty and NeoMTA Plus. Uppercase B (^B^) indicates significant difference (*p* < 0.05) between NeoPutty and MTA Angelus. Uppercase C (^C^) indicates significant difference (*p* < 0.05) between NeoMTA Plus and MTA Angelus. *ppm*, parts per million; *ppb* parts per billion

### MTT assay and cell migration

**Fig. 1:**
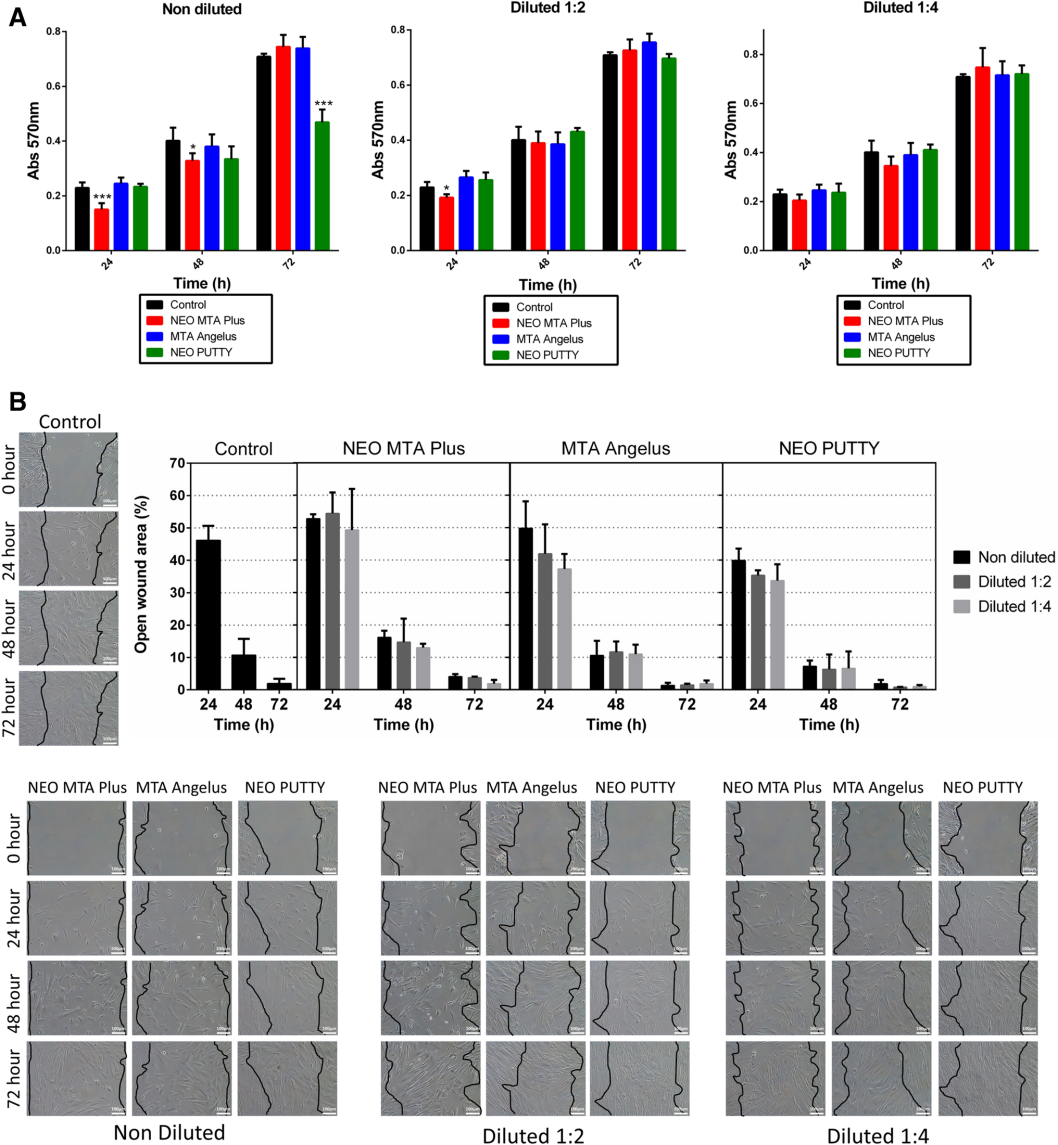
Cell viability and migration. **A** MTT assay for the evaluation of hDPCs after 24, 48, and 72 h of culture with the tested biomaterials and negative control (unconditioned medium). Asterisks designate significant differences compared to the control. *p < 0.05; **p < 0.01; ***p < 0.001. **B** The migration ability of hDPCs when cultured with the different material extracts was analyzed using a scratch wound assay. Images were captured every 24 h for 72 h using a phase-contrast microscope (× 100 magnification) and four standardized points were evaluated (0, 24, 48, and 72 h)

As shown in Fig. 1A, the control group maintained cell viability in all conditions. At 24 h of culture, 1:1 and 1:2 NeoMTA Plus exhibited a decreased cell viability compared to the control group (***p < 0.001; *p < 0.05, respectively). At 48 h of culture, undiluted NeoMTA Plus-treated cells evidenced a slight decrease in cell viability (*p < 0.05). At 72 h of culture, the viability of undiluted NeoPutty-treated cells was also reduced (***p < 0.001). However, 1:2 dilutions of all the tested materials exhibited a comparable cytocompatibility to that of the control group at 48 and 72 h of culture (*p* < 0.05). The same was observed for 1:4 dilutions of the tested materials at 24, 48, and 72 h of culture (*p* > 0.05).

Regarding cell migration, at 72 h of culture, NeoMTA Plus, MTA Angelus, and NeoPutty promoted wound healing without significant differences compared to the control group (Fig. 1B).

### Cell cytoskeleton labeling

**Fig. 2:**
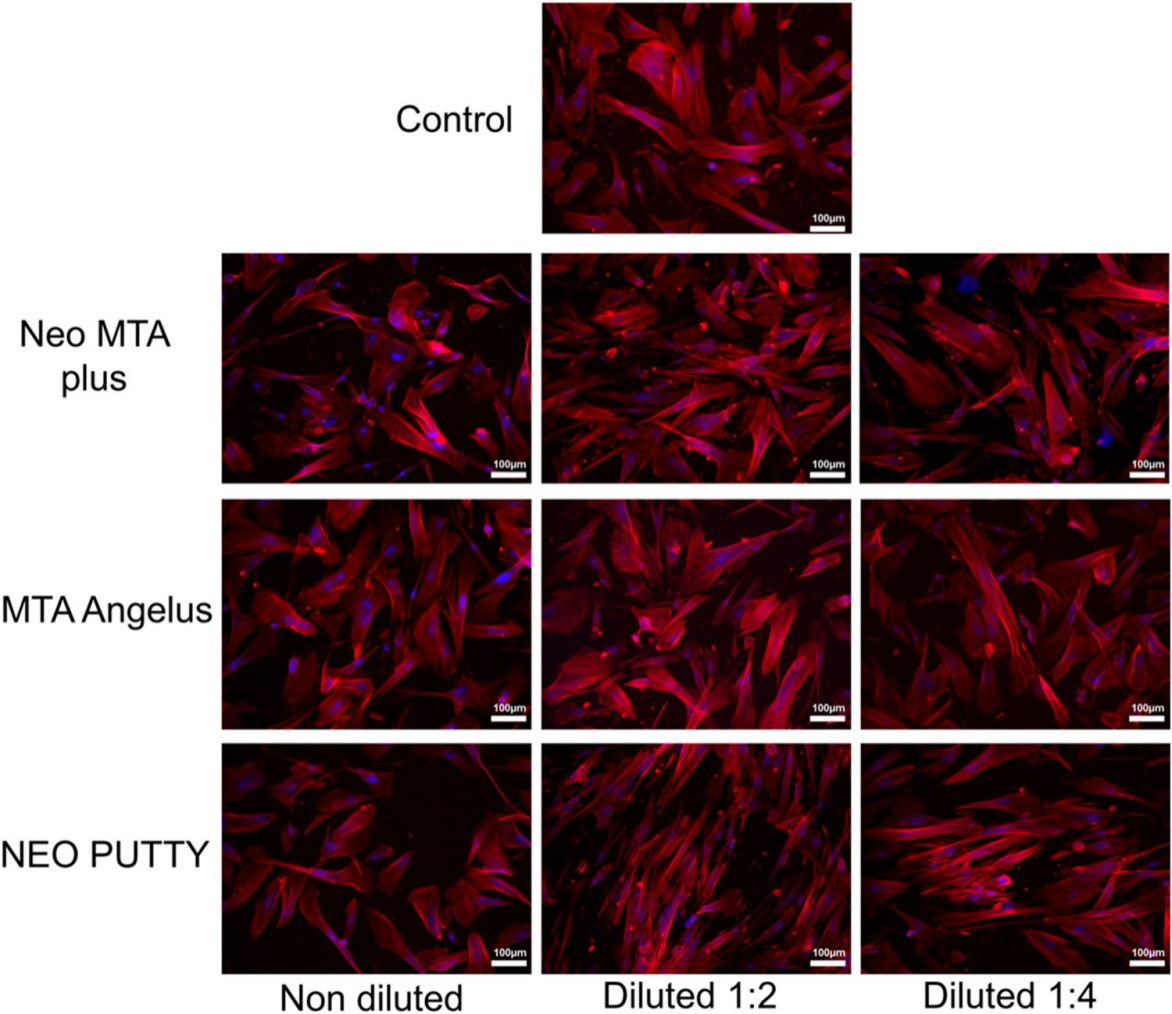
Cell cytoskeleton. Confocal images of hDPCs after treatment with NeoMTA Plus, NeoPutty, and MTA Angelus extracts. Blue fluorescence indicates cell nuclei, and red fluorescence, the actin cytoskeleton. Magnification: × 100. Scale bar = 100 μm

Phalloidin staining showed that cells treated with the tested material extracts exhibited a mesenchymal/fibroblastic cell morphology which was similar to the control group, mainly manifested by the regular display of F-actin, suggesting the adequate viability of hDPCs (Fig. 2).

### Cell attachment

**Fig. 3:**
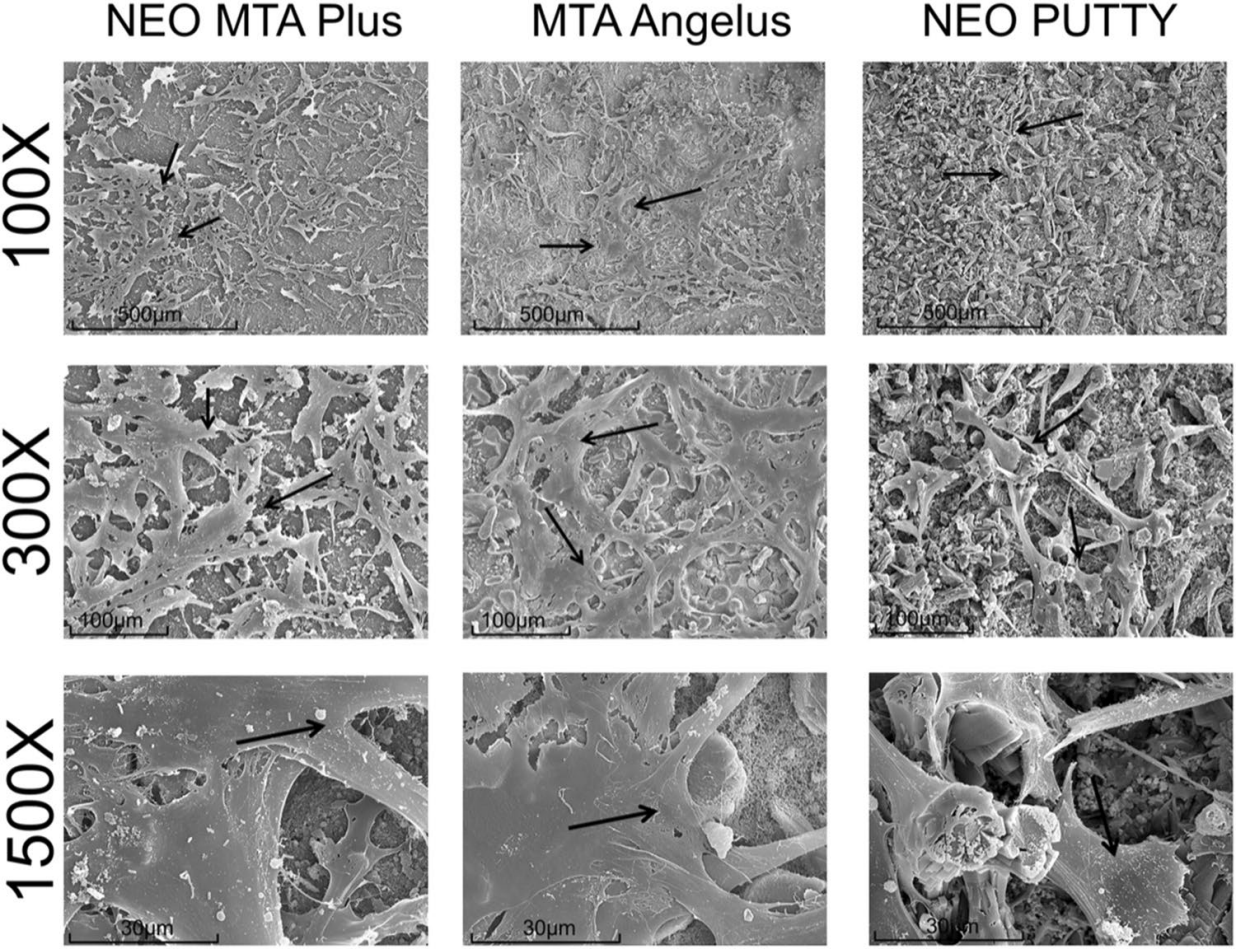
SEM analysis. SEM images show cell attachment on NeoMTA Plus, MTA Angelus, and NeoPutty disk surfaces. Black arrows indicate cells. Magnifications: × 100, × 300, and × 1500. Scale bars: 500 μm, 100 μm, and 30 μm

Representative scanning electron micrographs are shown in Fig. 3. The results showed abundant hDPCs firmly adhered to the surface of the tested materials and formed a network of interconnected cells, suggesting no cytotoxic effect.

### SEM–EDX analysis

**Fig. 4:**
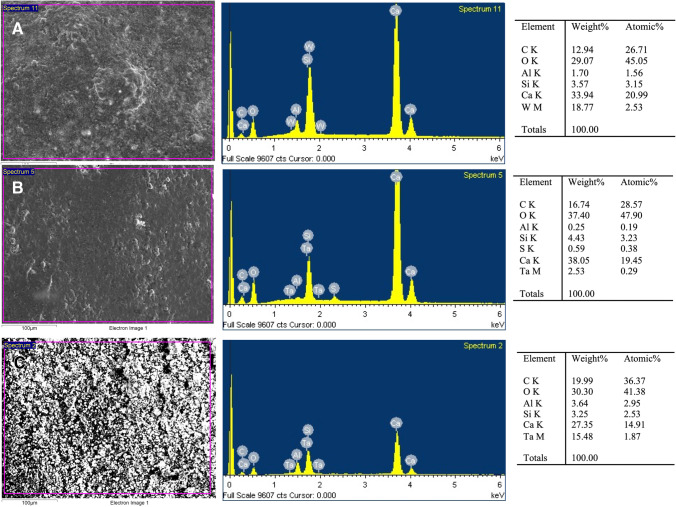
SEM–EDX analysis results for MTA Angelus (**A**), NeoMTA Plus (**B**), and NeoPutty (**C**) disks (*n* = 9). The first column presents SEM micrographs of each material (scale bar: 100 μm). The second column illustrates the EDX plots with the correspondent peaks detected. The third column classifies the list of elements present per material by weight and atomic weight

SEM–EDX analysis showed the chemical elements’ presence on the specimens’ surface in concordance to the material composition, as shown in Fig. 4 and Table 1. EDX analysis of NeoMTA Plus displayed a higher peak of Ca^2+^ than NeoPutty and MTA Angelus, whereas a high peak of tantalum (Ta^5+^) was also observed in NeoPutty. Parallelly, a moderate peak of Ta^5+^ was found in NeoMTA Plus. Finally, the concentrations of C, O, and Si were similar in all groups.

## Discussion

Ion-releasing materials are widely used in VPT, perforation repair, apexification, and other endodontic procedures [6, 19]. However, the available literature presents a wide variability in terms of the experimental methodology used to test the biological behavior of such materials [20]. Furthermore, there is little evidence about the new tantalum oxide (Ta_2_O_5_)-containing calcium silicate-based cement NeoPutty and its biological properties [13]. Variation in the composition of calcium silicate-based materials could lead to differences in their clinical behavior. For example, it has been suggested that differences in the radiopacifying agent may influence the biological properties of such materials, among others [21]. Accordingly, in this study, we aimed to assess the cytocompatibility of NeoPutty and to compare this property to that exhibited by MTA Angelus and NeoMTA Plus.

Pulp capping agents need to be cytocompatible in order to provide a biocompatible medium for dentin-pulp complex tissue repair. In other words, they cannot affect the viability of dental pulp cells negatively. Thus, hDPCs were chosen as the target cells for the in vitro assays. The alternative use of immortalized cells was discarded, since they are genetically modified and may exhibit clinically inappropriate toxic responses to the tested materials [22].

ICP-MS evidenced Ca^2+^ release and SEM–EDX revealed the presence of calcium in all the tested materials, as previously reported for other VPT materials [23]. Regarding ion release, the highest values of Ca^2+^ exhibited by MTA Angelus have been associated with its antimicrobial activity and mineralization potential. Since ion release depends on the material’s properties in terms of solubility, setting, and permeability to water [24], the lower release of Ca^2+^ from NeoMTA Plus and NeoPutty compared to MTA Angelus could be explained by existing differences in their hydration processes and setting reactions [25]. In addition, the release of calcium ions and calcium hydroxide deposition after hydration have been associated with the biological properties of VPT materials [26].

Accumulating evidence has demonstrated that ion release is a vital factor in generating cell responses, particularly cell metabolic activity and migration, and consequently enhances the pulp healing process [19, 27]. Also, these elements are of great importance in physiological processes and an essential part of living organisms. For example, magnesium and strontium stimulate mineralization activity, while zinc can improve overall bone quality with its antibacterial properties [28]. Parallelly, it has been reported that the addition of Ta^5+^ as a radiopacifying agent for ion-releasing materials does not influence their biological and physicochemical properties negatively and limits potential tooth discoloration [26].

It should be highlighted that the results from ICP-MS and SEM–EDX analyses showed differences between the superficial element distribution and the ion release in all the tested materials. For example, Al release was not detected by ICP-MS in MTA Angelus samples, but the presence of Al was evidenced in SEM–EDX analysis. The most feasible explanation is the lack of Al release due to the setting of the material. Differences were also observed between the release of Sr or Ba. Together with Al, these trace elements were measured in ppb (parts per billion), since they were released in very low proportions. Interestingly, the presence of these elements is not reflected in the composition of the tested materials (Table 1). This can be explained by the presence and proportion of a series of components which are often regarded as confidential business information (CBI) and thus are not reflected in the composition from the materials’ respective safety data sheets (SDS). The implications of the release of these ions in the behavior of the tested materials are yet to be elucidated.

Regarding the results from cell viability assays, in general terms, the undiluted (1:1) NeoPutty and NeoMTA Plus extracts exhibited a decreased cell viability. Although Ca2 + is an essential regulator of several intracellular processes, excessive intracellular accumulation of Ca2 + and the high alkalinity of the culture medium may be related to mitochondrial dysfunction and consequently a reduced cell viability [29].

However, 1:2 and 1:4 eluents of the tested materials resulted in comparable cell viability to that of the control group. The increased cytocompatibility of calcium silicate-based materials as more diluted has also been reported in previous in vitro studies [7, 30]. The use of three dilutions (1:1, 1:2, and 1:4) was performed to simulate the clinical conditions, in which the tested materials can be placed on the remaining dentin thicknesses of 0.01 to 0.25 mm or directly on pulp exposures. Therefore, the concentration of the material that reaches viable pulp tissue may differ.

Consistent with our findings, previous studies have reported that, to a certain degree, eluents of mineral trioxide aggregate (MTA) and tantalum oxide (Ta2O5)-containing pulp capping agents promoted cell viability [16, 31]. Conversely, other vital pulp materials such as White-MTAFlow showed low cell viability values in vitro studies [25].

Several studies have reported enhanced cell migration activity induced by VPT materials [32, 33]. Although the ability of VPT materials to stimulate hDPC migration has been shown in 2D culture, our findings showed that NeoMTA Plus, MTA Angelus, and NeoPutty exhibited no statistically significant differences compared to the untreated control, irrespective of the concentrations used. These results agree with a previous report in which the cytotoxicity of NeoMTA 2 and NeoMTA Plus were compared with the Portland cement-based material MTA (Angelus, Londrina, PR, Brazil). Like the present study, all materials evidenced adequate cell migration [7].

Numerous studies have reported the role of biomaterials in augmenting essential epigenetic functions via the modification of the cell’s actin cytoskeleton, resulting in increased or decreased cell attachment [34, 35]. Thus, this acts as a direct indicator of their biocompatibility. In the present study, the morphological characteristics and adhesion of hDPCs exposed to VPT materials were similar to those of the control group (cells grown in the absence of material extracts), exhibiting high cell density and ability to spread. Hence, these results suggest the cytocompatibility of the tested materials. However, it should be highlighted that the results from these assays are descriptive non-statistical morphological observations and are consequently left to the interpretation of the investigators. Nevertheless, these types of biological assays are commonly used among in vitro studies on the biological properties of calcium silicate-based endodontic materials [17, 26, 36], as a complement to the quantifiable biological assays, i.e., viability and proliferation/migration assays.

SEM–EDX analysis detected aluminum and sulfur peaks for NeoMTA, but not sulfur peaks for NeoPutty, differing from that reported by the manufacturer. In addition to calcium silicate, NeoPutty incorporates calcium aluminate, tantalite, calcium aluminate, calcium sulfate, proprietary organic liquid, and stabilizers in its composition. Calcium aluminate has been shown to support the acquisition of osteogenic cell phenotypes in vitro [37]. In addition, calcium aluminate-containing materials have shown adequate biocompatibility after subcutaneous implantation in rats [38]. As expected, MTA Angelus did not show tantalum peaks, since tantalum oxide is not reported in its composition. On the other hand, all VPT materials showed calcium peaks due to the calcium present in their composition. The minor differences observed may be due to EDX’s elemental mapping, which only shows the distribution of the elements on the sample’s surface.

To date, scientific evidence regarding the biological properties of NeoPutty remains limited. In a previous in vitro study, it was reported that NeoPutty exhibited a higher biocompatibility than another calcium silicate-based material (EndoSequence BC RRM putty (Brasseler, USA)) in contact with human dental pulp stem cells [16]. In the present study, the similar biocompatibility of NeoPutty to its predecessor (NeoMTA Plus) and the classic gold standard MTA Angelus towards hDPCs is elucidated. Altogether, both studies coincide with regard to the cytocompatibility of NeoPutty. However, due to the in vitro nature of the discussed results, the extrapolation of the observed behaviors to the clinical setting remains in a preliminary stage. Further in vitro studies in different conditions, animal studies, and clinical trials are needed to reinforce existing evidence.

## Conclusions

The results from the present in vitro study elucidate the cytocompatibility of NeoPutty, NeoMTA Plus, and MTA Angelus towards human dental pulp cells. Further studies on different conditions, i.e., animal models or clinical studies, are needed to confirm the suitability of NeoPutty as a pulp capper for vital pulp treatment procedures.
